# Effect of radiotherapy on the expression of cardiovascular disease-related miRNA-146a, -155, -221 and -222 in blood of women with breast cancer

**DOI:** 10.1371/journal.pone.0217443

**Published:** 2019-05-31

**Authors:** Roser Esplugas, Meritxell Arenas, Noemí Serra, Montserrat Bellés, Marta Bonet, Marina Gascón, Joan-Carles Vallvé, Victoria Linares

**Affiliations:** 1 Physiology Unit, School of Medicine, IISPV, Rovira i Virgili University, Reus, Spain; 2 Laboratory of Toxicology and Environmental Health, School of Medicine, IISPV, Rovira i Virgili University, Reus, Spain; 3 Radiation Oncology Department, Sant Joan University Hospital, IISPV, Rovira i Virgili University, Reus, Spain; 4 Radiation Oncology Unit, Miguel Servet University Hospital, Zaragoza, Spain; 5 Research Unit on Lipids and Atherosclerosis, Sant Joan University Hospital, IISPV, Rovira i Virgili University, Reus, Spain; Université de Picardie Jules Verne, FRANCE

## Abstract

Breast cancer (BC) is one of the most important neoplasias among women. Many patients receive radiotherapy (RT), which involves radiation exposure of the thoracic zone, including the heart and blood vessels, leading to the development of cardiovascular disease (CVD) as a long-term side effect. The severity of CVD-related pathologies leads research on assessing novel CVD biomarkers as diagnostic, prognostic or therapeutic agents. Currently, the possible candidates include blood microRNAs (miRNAs). Previous studies have supported a role for miRNA-146a, -155, -221, and -222 in the progression of CVD. Our purpose was to evaluate the RT-induced modulation of the expression of these miRNAs in the blood of women with BC. Pre-RT control and post-RT blood samples were collected, and after miRNA isolation and reverse transcription, the levels of the selected miRNAs were measured by real-time PCR. Our results showed that miRNA-155 exhibited the lowest expression, while miRNA-222 exhibited the highest expression, followed by miRNA-221. The expression of each individual miRNA was positively correlated with that of the others both pre-RT control and post-RT and inversely correlated with age before RT. Furthermore, RT promoted the overexpression of the selected miRNAs. Their levels were also affected by CVD-linked clinical parameters, treatment and BC side. Modulation of the expression of the selected miRNAs together with other risk factors might be associated with the development of future cardiovascular pathologies. Further confirmatory studies are needed to assess their potential as possible biomarkers in the progression of or as therapeutic targets for RT-induced CVD in BC patients.

## Introduction

Breast cancer (BC) is one of the most important neoplasias among women. In Spain, 1 in 8 women will develop BC, and approximately 26,000 new cases per year are diagnosed [1]. According to the Instituto Nacional de Epidemiología, there were 6,213 BC deaths in 2014 [2]. Currently, fewer cases in UE and North America are diagnosed because of both early detection and efficient systemic therapies. Nevertheless, it is still the most common cause of death from cancer in less developed countries and the second cause in more developed countries, subsequent to lung cancer [3,4].

The treatment of BC involves a combination of therapies such as surgery, chemotherapy, hormonotherapy, targeted therapy and radiotherapy (RT) [5]. RT can be omitted in some patients with a low-risk profile, which reduces the risk of local recurrence and mortality [6]. RT treatment involves radiation exposure of the thoracic zone, including the heart and blood vessels. Despite RT-related benefits, epidemiological data support a relationship between RT and cardiovascular disease (CVD) [7–9].

Patients with left-sided BC exhibit greater cardiotoxicity than those with right-sided tumours due to RT exposure to the heart and left coronary artery [10–12]. In a retrospective study, Darby et al. [12] observed that for every 1 Gy increase in radiation to the heart, the risk of cardiac damage increased 7.4% in patients with left-sided BC. These authors also reported similar results following ionizing radiation to the heart during RT of BC. Cheng et al. [13], who examined 1,191,371 patients from 39 studies, supported that RT applied to patients with left-sided breast tumours caused major coronary heart failure and cardiac death compared with patients with right-sided BC. They also stated that the risk of CVD begins within the first ten years after RT and that heart mortality increases in the second and third decades. Although the RT-CVD association is lower than that established by risk factors such as smoking, hypertension, diabetes or hyperlipidaemia, the risk of death due to CVD after this therapy exceeds the increase in RT-contributed survival. The CVDs associated with RT in BC patients are coronary heart disease [13,14] and atherosclerosis [15].

The severity of these CVD-related pathologies leads research on assessing novel CVD biomarkers. Currently, the possible candidates as suitable biomarkers in diagnostic, prognostic or therapeutic agents of this pathogenesis are microRNAs (miRNAs) [16–19].

miRNAs are small noncoding RNAs of approximately 22 nucleotides in length that act as posttranscriptional regulators, thus modulating gene expression [20,21]. Approximately 1,500–2,000 human miRNAs have been identified, and it is estimated that over 60% of protein-coding genes are directly regulated by miRNAs. One miRNA targets several mRNAs, and one mRNA could be regulated by different miRNAs. Furthermore, miRNAs are ubiquitously expressed, non-cell-specific and can be released into the circulation through microvesicles (i.e., exosomes and apoptotic bodies), lipoproteins (i.e., HDL), or as a protein complex (i.e., RISC and necrotic tissue) [22]. Blood miRNAs are useful as biomarkers for disease diagnosis because the extraction method is noninvasive, they are sensitive, disease-specific, and stable in body fluids for a long time and, finally, freeze-thaw cycles do not appear to affect their structure [23–25]. In fact, blood miRNAs have recently become attractive biomarkers in pathologies including CVD due to their role as disease regulators [26,27].

Previous studies have supported a role for miRNA-146a, -155, -221, and -222 in the progression of CVD, mainly by regulating inflammation, oxidative stress, apoptosis, and angiogenesis in atherosclerotic plaques [28–31]. It is supported that all these miRNAs are overexpressed in human atherosclerotic lesions [32–34]. miRNA-146a is an atheroprotective and anti-inflammatory miRNA that acts in oxidized LDL-activated macrophages, reducing lipid uptake and cytokine release [28,35], but its absence paradoxically suppresses atherosclerosis development in mice [32]. Moreover, miRNA-155 regulates immune and inflammatory processes and induces lipid uptake in monocytes and macrophages [29,36]. Finally, both miRNA-221 and 222 exhibit antiproliferative and proapoptotic actions in endothelial cells, whereas they promote proliferation and migration and inhibit apoptosis in smooth muscle cells [37,38].

Therefore, the aim of our study was to evaluate the expression of these miRNAs in the blood of pre-RT control and post-RT patients with BC.

## Materials and methods

### Participant treatment

The study was approved by the Ethics Committee (Institutional Review Board) of the Hospital Universitari de Sant Joan (Reference: CEIM 014/2017). All patients signed a written informed consent according to the declaration of Helsinki and a written informed consent for publication.

In the study, 136 women (mean age: 56 years, range: 27–84 years) diagnosed with BC were included. They were patients at the Department of Radiation Oncology of Hospital Sant Joan (Reus, Spain). All patients had a Karnofsky Index <70 and were classified as 0 or 1 on the Eastern Cooperative Oncology Group scale [39]. The protocol for metastatic BC patients (2.94%) was to proceed to RT following complete response of the metastases to primary systemic treatment [39].

The applied treatment for these women was as follows. First, the tumour was extirpated by surgery. Then, according to their risk factor status, they received RT alone or RT combined with other therapies. On the one hand, some patients received adjuvant chemotherapy (55.2%) and/or targeted therapy (17.6%), which were administered postsurgery, for approximately 4 or 5 months and was concluded between 1 and 2 months before RT. On the other hand, adjuvant hormone therapy commenced 1 to 2 months postsurgery and was usually administered simultaneously with RT (78.7% of patients).

### Radiotherapy schedule

All patients received RT for approximately 2 months. The radiation scheduled could be normofractionated RT (50 Gy at 2 Gy/day on the affected breast and 16 Gy at 2 Gy/day on the tumour bed for 5 days/week) or hypofractionated RT (40 Gy at 2.67 Gy/day for 5 days/week). The dose rate of all schedules was 4.5 Gy/min (400 MU/min) [40]. Additionally, some patients received irradiation to the regional lymph nodes depending on risk factor status [41]. During RT, a weekly acute toxicity assessment was performed using the criteria of the Radiation Therapy Oncology Group and the European Organization for Research and Treatment of Cancer [42].

### Biological samples

Blood samples of the patients were collected at two time-points: prior to RT (named as pre-RT control) and one month after the conclusion of RT (named as post-RT). Samples were collected into “PAXgene Blood RNA” tubes (Qiagen, Madrid, Spain) allowing the stabilization of intracellular miRNA, and they were stored at -80°C until use.

### miRNA isolation

For the miRNA isolation, 2mL of blood samples were centrifuged for 10min at 4,000 g. Then, supernatant was discarded and pellet was resuspended with 2mL PBS. After 10min at 4,000g centrifugation, supernatant was discarded and the next steps were performed by following the miRCURY RNA Isolation Kit (Exiqon, BioNova, Madrid, Spain). Briefly, the pellet was resuspended with 350μL of lysis buffer mixed with β-mercaptoethanol (100:1 ratio). Then, 200μL of 100% ethanol was added and mixed by vortex. The content was transferred onto a column and centrifuged 1min at 3,500g. The flowthrough was discarded and the column was washed three times by adding 400μL of Wash Solution and subsequently centrifuging 1min at 14,000g. Then, the flowthrough was discarded and the column was dry off by centrifuging 2min at 14,000g. Finally, 50μL of Buffer Elution was added onto the column and sample was centrifuged twice for 2min at 200g and 1min at 14,000g. The flowthrough containing miRNA was stored into RNA-free tubes at -80°C until use.

### cDNA synthesis

cDNA was synthesized using the miRCURY LNA Universal RT microRNA PCR, Polyadenylation and cDNA synthesis kit II (Exiqon, BioNova, Madrid, Spain). Briefly, 2μL of miRNA sample was mixed with 1μL Reverse Transcriptase enzyme, 2μL Reaction buffer and 5μL H_2_O. Then, GeneAmp PCR System 2700 Thermal Cycler (Applied Biosystems, Thermo Fisher Scientific, Waltham, Massachusetts, USA) was employed for the sample incubation. Using the thermocycler program, the miRNAs samples were reversing transcript for 60min at 42°C, the Reverse Transcriptase was inactivated for 5min at 95°C and the samples containing cDNA were maintained at 4°C and finally stored at -20°C.

### Real-time PCR

To determine miRNA levels, real time PCR assays were performed. The assessed miRNAs were miRNA-146a, -155, -221 and -222. Levels of target miRNAs were normalized employing the snRNA-U6 as housekeeper. Briefly, the reactions were performed in a final reaction volume of 10μL, adding 3.5μL cDNA sample (previously diluted 1:40 with RNase-free water) and 6.5 μL mix containing 5μL ExiLENT SYBR Green Master Mix (Exiqon, BioNova, Madrid, Spain), 1 μL tested Primer (miRCURY LNA uniRT PCR primer mix, Exiqon), 0.2μL ROX (Passive reference RT-PARE-03, Exiqon, BioNova, Madrid, Spain) and 0.3 μL RNase-free water. Negative controls were also run for each assay. Real-time reactions were run by use of a 7900 HT Fast Real-Time PCR System (Applied Biosystems, Thermo Fisher Scientific, Waltham, Massachusetts, USA), performing dsDNA denaturation activation for 10min at 95°C and 40 cycles of 10 s at 95°C followed by 1min at 60°C for amplification. Finally, results were analysed using the 2.4 SDS Software (Applied Biosystems, Thermo Fisher Scientific, Waltham, Massachusetts, USA) and RQ Manager 1.2.1 (Applied Biosystems, Thermo Fisher Scientific, Waltham, Massachusetts, USA).

### Statistical analysis

For the statistical analysis, 2^-DCt^ of selected miRNAs was calculated for each sample. DCt was calculated as Cycle threshold (Ct) of each miRNA–Ct of snRNA-U6. Then, in order to normalize the variable we transformed into log (2^-DCt^) and in order to positivize it we added a 3 units constant to obtain log (2^-DCt^) + 3. Statistics of transformed variable log (2^-DCt^) + 3 did not differ from log (2^-DCt^) ones. First, ANOVA test was used to evaluate differences between their expressions and percentage of increase was calculated for each miRNA in comparison to the lower expressed one. Then, correlations between miRNAs expression and age at diagnose of patients were assessed employing Pearson correlation coefficient. Moreover, paired t-test was performed in order to assess the differences between pre-RT and post-RT expressions in blood of the selected miRNAs and their percentage of increase was calculated. Finally, single factors ANOVA tests were executed to evaluate the differences in their expression depending on CVD-linked clinical parameters, received treatment and BC side. The level of statistical significance for all tests was established at p<0.05. All the calculations were performed with IBM SPSS Statistics (SPSS version 25 for Windows, USA).

## Results

### Clinical characteristics of BC patients

The main clinical characteristics of the BC patients are shown in Table 1. On the one hand, when recluded before RT, some women presented CVD-related diseases, such as diabetes mellitus (8.1%), arterial hypertension (25.7%), dyslipidaemia (27.2%), cardiac pathology (2.9%) or hypothyroidism (7.4%). Furthermore, the majority were postmenopausal. On the other hand, prevalent tumour molecular classifications were luminal A and B types. Moreover, 55.1% of women received chemotherapy, 78.7% of women received hormonal therapy, and targeted therapy was applied to only 17.6% of patients. Lastly, the affected BC side was similarly right or left, whereas only 1.5% of women presented bilateral BC.

**Table 1:** Characteristics of BC patients.

Variable		Total	Frequency (%)
**Diabetes mellitus**		11	8.1
**Arterial hypertension**		35	25.7
**Dyslipidemia**		37	27.2
**Cardiac pathology**		4	2.9
**Hypothyroidism**		10	7.4
**Menopause state**			
** **	**Premenopausal**	29	21.3
** **	**Perimenopausal**	20	14.7
** **	**Postmenopausal**	85	62.5
**Tumor molecular classification**			
** **	**Luminal A**	40	29.4
** **	**Luminal B**	50	36.8
** **	**Her2 positive**	27	19.9
** **	**Triple negative**	17	12.5
**Ki67 antigen in tumor biopsy**			
** **	**<15**	48	35.3
** **	**15–50**	62	45.6
** **	**>50**	19	14.0
**HER2 receptor in tumor biopsy**			
** **	**negative**	107	78.7
** **	**positive**	26	19.1
**Estrogen receptors**			
** **	**negative**	22	16.2
** **	**positive**	113	83.1
**Progesterone receptors**			
** **	**negative**	46	33.8
** **	**positive**	89	65.4
**Chemotherapy**		75	55.1
**Hormone therapy**		107	78.7
**Targeted therapy**		24	17.6
**BC location**			
** **	**Right**	66	48.5
** **	**Left**	68	50.0
** **	**Bilateral**	2	1.5

BC, breast cancer; HER2, human epidermal growth factor receptor 2.

### Expression of miRNA-146a, -155–221 and -222 at pre-RT control and post-RT

**Fig 1:**
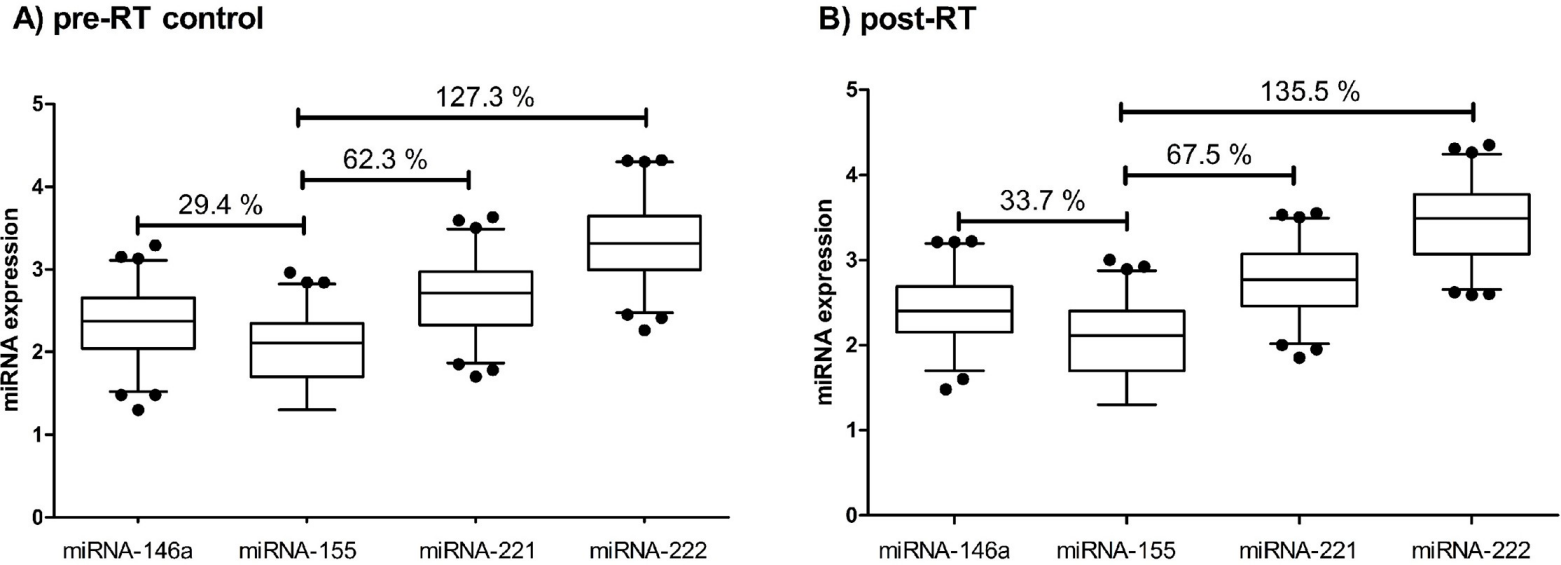
**Expression of miRNA-146a, -155, -221 and -222 in blood of BC patients at pre-RT control (A) and post-RT (B) treatment.** miRNA expression was calculated as log(2^-DCt^)+3. Results are expressed as percentile 2.5–97.5. Percentage of expression increase in comparison to miRNA-155 is indicated (%).

The levels of miRNA-146a, -155–221 and -222 in the blood of women with BC are shown in Fig 1A–1D. In the current study, miRNA-155 presented the lowest expression both pre-RT control and post-RT. When compared to this miRNA, the expression of miRNA-146a, -221 and -222 before RT was significantly higher (29.4%, 62.3% and 127.3%, respectively, p< 0.0001) (Fig 1A). In addition, similar differences in expression were maintained after RT, with significant increases of 33.7%, 67.5% and 135.5%, respectively (p< 0.0001) (Fig 1B).

### Correlations of miRNA-146a, -155, -221 and -222 expression at pre- RT control and post-RT

Table 2 shows the correlations between miRNA expression in the blood before and after RT in women with BC. The results revealed a significant positive correlation between miRNA expression both pre-RT control and post-RT; the Pearson correlation coefficient (*r*) was above 0.882 for all bivariate correlations.

**Table 2:** Correlations between expression of selected miRNAs in blood of BC patients at pre-RT control and post-RT.

	Pre-RT control			Post-RT		
	miRNA-146a	miRNA-155	miRNA-221	miRNA-146a	miRNA-155	miRNA-221
**miRNA-155**	0.927**			0.924**		
**miRNA-221**	0.900**	0.917**		0.896**	0.909**	
**miRNA-222**	0.890**	0.910**	0.930**	0.882**	0.906**	0.922**

BC, breast cancer; RT, radiotherapy; miRNA, microRNA.

miRNA expression was calculated as log(2-DCt)+3. Pearson correlation coefficient (r) is shown.

**means significant differences at p<0.01 (bilateral).

**Fig 2:**
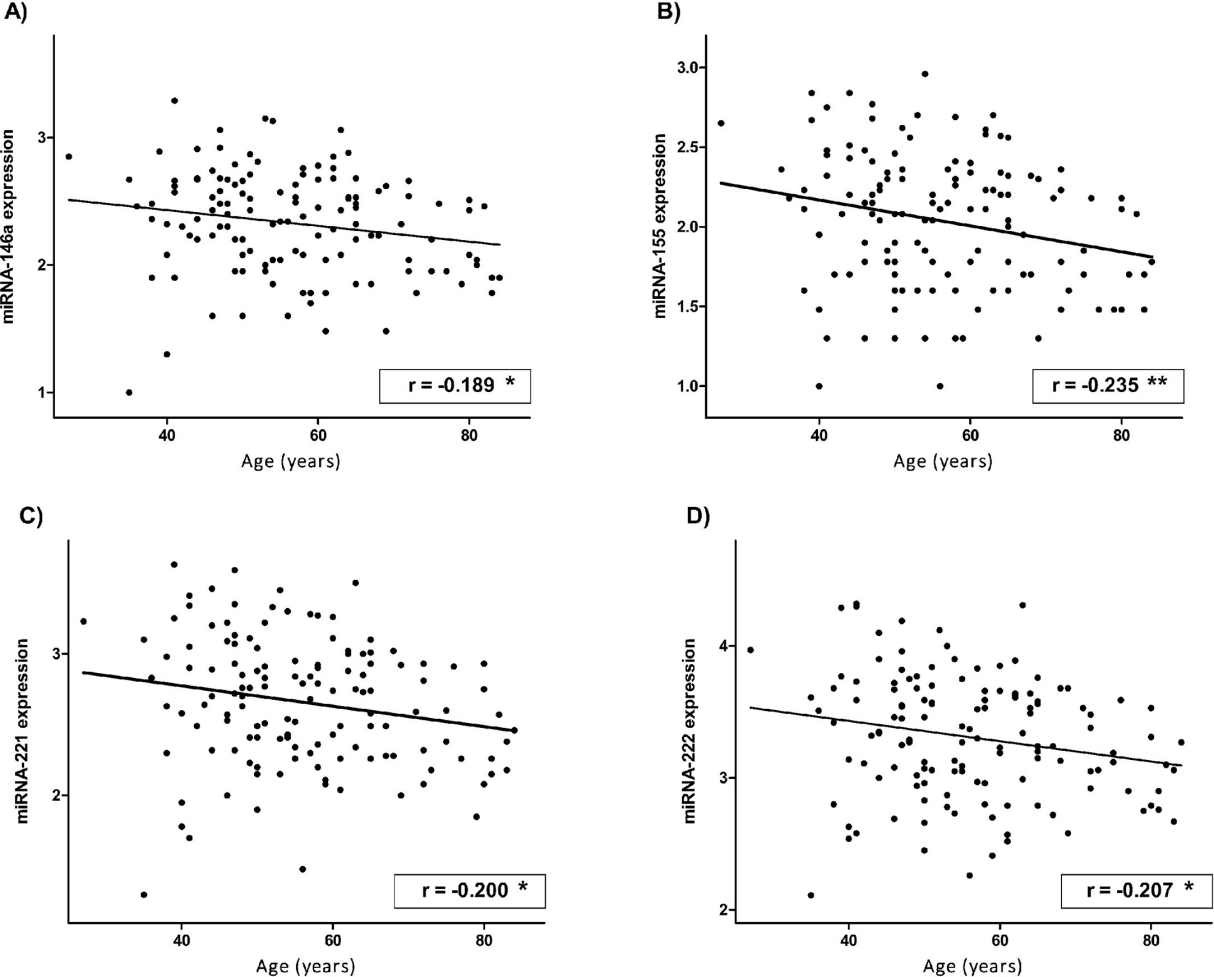
**Correlations of the expression of miRNA-146a (A), -155 (B), -221 (C) and -222 (D) in blood of BC patients with age at diagnose at pre-RT control.** miRNA expression was calculated as log(2^-DCt^)+3. Pearson correlation coefficient (r) is shown. -means inverse correlation. *means significant differences at p<0.05 (bilateral). **means significant differences at p<0.01 (bilateral).

Moreover, the expression of all studied miRNAs pre-RT was inversely correlated with age at diagnosis (Fig 2). The results also showed that after RT, this significant correlation was lost, although the same tendency persisted (S1 Table of Supporting information).

### Comparison of the expression of each miRNA between pre- RT control and post-RT time points

**Fig 3:**
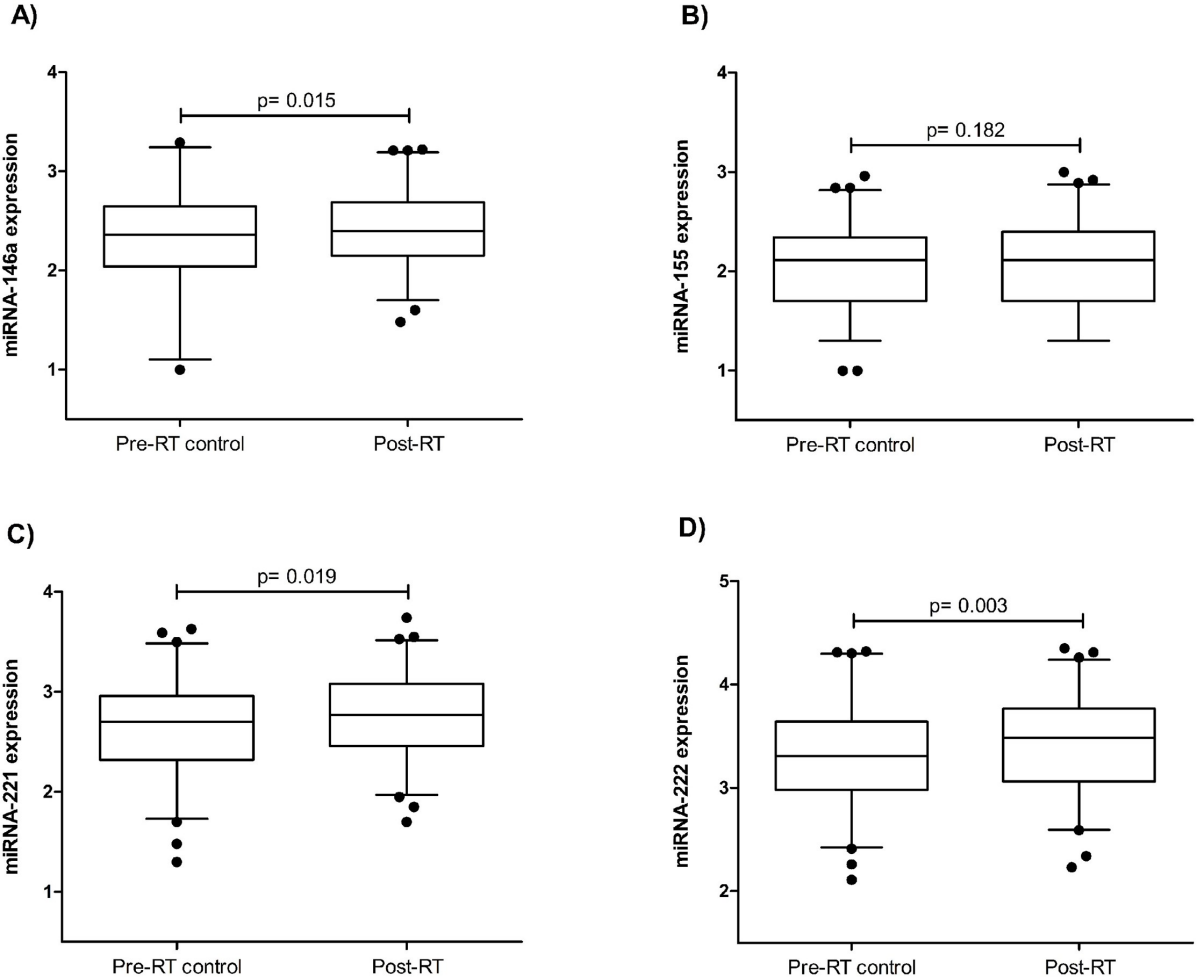
**Comparison of each blood miRNA (miRNA-146a (A), -155 (B), -221 (C) and -222 (D)) of BC patients between pre-RT control and post-RT time-points.** miRNA expression was calculated as log(2^-DCt^)+3.p value indicates differences between groups.

The expression of the selected miRNAs in the blood before and after RT in women with BC is depicted in Fig 3 (A-D). We observed that RT treatment induced an increase in the expression of the selected miRNAs whose levels were higher post-RT than pre-RT control. While miRNA-222 had the greatest statistically significant increase (12%, p = 0.003) (Fig 3D), miRNA-146a (Fig 3A) and -221 (Fig 3C) also increased significantly (7.6%, p = 0.015 and 9%, p = 0.019, respectively). Furthermore, miRNA-155 decreased by 3.8%, without statistical significance (p = 0.182) (Fig 3B).

### Evaluation of miRNA-146a, -155, -221 and -222 expression at pre- RT control and post-RT considering CVD-related clinical parameters, treatment and BC side

We assessed whether the expression of the selected miRNAs was associated with the radiation schedule, and we found no differences between miRNA levels in women who received normofractionated RT or hypofractionated RT at either time point (S2 Table of Supporting information).

Table 3 shows the levels of miRNA-146a, -155, -221 and -222 in the blood of BC patients pre-RT control and post-RT stratified by dyslipidaemia, hypothyroidism, chemotherapy, targeted therapy and BC side. On the one hand, the expression of all miRNAs was significantly associated with dyslipidaemia both pre-RT control (miRNA-146a; p = 0.023, miRNA-155; p = 0.016, miRNA-221; p = 0.023, miRNA-222; p = 0.005) and post-RT (miRNA-146a; p = 0.023, miRNA-155; p = 0.033, miRNA-221; p = 0.018, miRNA-222; p = 0.009); dyslipidaemic patients had significantly lower levels than nondyslipidaemic patients. Similarly, hypothyroid women presented significantly lower levels than nonhypothyroid women before (miRNA-146a; p = 0.026, miRNA-155; p = 0.036, miRNA-222; p = 0.015) and after (miRNA-146a; p = 0.028, miRNA-155; p = 0.034, miRNA-221; p = 0.017, miRNA-222; p = 0.009) RT, excluding miRNA-221 pre-RT. In addition, the expression of some miRNAs was significantly associated with treatment. Women who received chemotherapy showed significantly increased levels of all the selected miRNAs pre-RT control (miRNA-146a; p = 0.003, miRNA-155; p = 0.000, miRNA-221; p = 0.000, miRNA-222; p = 0.001) and miRNA-155 (p = 0.025) and -221 post-RT (p = 0.030) compared to those not treated with chemotherapy. Moreover, regarding targeted therapy, the expression of miRNA-155, -221 and -222 prior to RT was significantly higher in treated women than in nontreated women (miRNA-155; p = 0.045, miRNA-221; p = 0.003, miRNA-222; p = 0.005). In contrast, the expression of these miRNAs did not differ significantly between women who received hormonal therapy and those in whom this therapy was not applied (S2 Table of Supporting information). Finally, the BC side was also significantly associated with miRNA expression. A tendency toward higher levels of all miRNAs was observed in women with left-sided BC compared to those with right-sided BC, which was significant for miRNA-146a both pre-RT control (p = 0.033) and post-RT (p = 0.025) and for miRNA-221 pre-RT control (p = 0.021).

**Table 3:** Expression of selected miRNAs expression in blood of BC patients at pre-RT control and post-RT depending on CVD-linked clinical parameters, received treatment and BC side.

	Pre-RT control			Post-RT		
**Dyslipidemia**	**No** **(71.3%)**	**Yes** **(27.2%)**	**p value**	**No** **(71.3%)**	**Yes** **(27.2%)**	**p value**
** miRNA-146a**	2.38±0.043	2.20±0.057	0.023	2.45±0.037	2.28±0.070	0.023
** miRNA-155**	2.08±0.044	1.88±0.064	0.016	2.12±0.041	1.94±0.075	0.033
** miRNA-221**	2.71±0.048	2.51±0.060	0.023	2.80±0.040	2.61±0.074	0.018
** miRNA-222**	3.37±0.049	3.12±0.063	0.005	3.49±0.042	3.26±0.082	0.009
**Hypothyroidism**	**No** **(91.2%)**	**Yes** **(7.4%)**	**p value**	**No** **(91.2%)**	**Yes** **(7.4%)**	**p value**
** miRNA-146a**	2.35±0.037	2.06±0.1	0.026	2.43±0.034	2.15±0.144	0.028
** miRNA-155**	2.05±0.039	1.76±0.10	0.036	2.07±0.037	1.80±0.141	0.034
** miRNA-221**	2.68±0.041	2.38±0.10	0.450	2.77±0.036	2.45±0.146	0.017
** miRNA-222**	3.33±0.042	2.96±0.10	0.015	3.46±0.039	3.07±0.173	0.009
**Chemotherapy**	**No** **(39%)**	**Yes** **(55.1%)**	**p value**	**No** **(39%)**	**Yes** **(55.1%)**	**p value**
** miRNA-146a**	2.20±0.053	2.42±0.047	0.003	2.34±0.045	2.45±0.050	0.119
** miRNA-155**	1.86±0.054	2.14±0.048	0.000	1.97±0.053	2.15±0.051	0.025
** miRNA-221**	2.46±0.056	2.78±0.050	0.000	2.65±0.053	2.81±0.050	0.030
** miRNA-222**	3.13±0.055	3.41±0.055	0.001	3.34±0.055	3.49±0.055	0.069
**Targeted therapy**	**No** **(78.7%)**	**Yes** **(17.6%)**	**p value**	**No** **(78.7%)**	**Yes** **(17.6%)**	**p value**
** miRNA-146a**	2.3±0.038	2.47±0.091	0.054	2.41±0.036	2.38±0.094	0.709
** miRNA-155**	2.00±0.041	2.19±0.088	0.045	2.08±0.040	2.08±0.098	0.940
** miRNA-221**	2.60±0.041	2.89±0.093	0.003	2.75±0.040	2.73±0.089	0.803
** miRNA-222**	3.25±0.042	3.53±0.106	0.005	3.44±0.043	3.38±0.096	0.563
**BC side**	**right** **(48.5%)**	**left** **(50%)**	**p value**	**right** **(48.5%)**	**left** **(50%)**	**p value**
** miRNA-146a**	2.24±0.053	2.42±0.044	0.033	2.32±0.048	2.50±0.044	0.025
** miRNA-155**	1.96±0.054	2.11±0.050	0.133	2.00±0.052	2.14±0.050	0.133
** miRNA-221**	2.55±0.058	2.76±0.048	0.021	2.66±0.050	2.83±0.049	0.053
** miRNA-222**	3.22±0.060	3.39±0.052	0.083	3.37±0.056	3.48±0.053	0.568

BC, breast cancer; RT, radiotherapy; miRNA, microRNA.

Mean ± SEM of the expression (log(2^-DCt^)+3) of selected miRNAs is shown. Statistics: Single factor ANOVA test. p value indicates differences between groups.

## Discussion

In the present study, we evaluated the expression of miRNA-146a, -155, -221 and -222, which are involved in the development of CVD, in the blood of both pre-RT control and post-RT samples from women with BC. Our results showed that at both time points, miRNA-222 had the highest expression, followed by miRNA-221. Both miRNAs, miRNA-221 and 222, are involved in the regulation of development and progression of atherosclerosis, exhibiting antiproliferative and proapoptotic actions in endothelial cells [37]. In contrast, they play an opposite role in smooth muscle cells, inducing proliferation and migration and inhibiting apoptosis [38]. On the other hand, the expression of miRNA-155 was the lowest in both the pre-RT control and post-RT blood samples. This observation is in accord with Gezer et al. [43], who indicated that the circulating miRNA-221 levels were higher than those of miRNA-155 in BC patients. Furthermore, miRNA-155 was recently considered below the detection limit by Alunni-Fabbroni et al. [44], who was not able to detect the expression of this miRNA in the circulation of BC patients. In addition, our results showed that the expression of each of the four selected miRNAs was positively correlated with that of the others both pre-RT control and post-RT. Their correlation could be supported by a linked regulation of their expression, as a crosslink between these miRNAs has been described in several pathways in which they are involved, such as inflammatory processes that regulate atherosclerotic processes [31,45,46]. Similarly, Baldeon et al. [47] found a correlation between circulating miRNA-146 and -155 in diabetics and in healthy patients.

We reported that the expression of the selected miRNAs was inversely correlated with the age at diagnosis of patients before RT. Thus, it was observed that with age, the levels of the selected miRNAs decreased significantly. Human ageing is associated with an increase in age-related diseases, such as cancer, diabetes mellitus and CVD [48,49]. A number of investigations have suggested that longevity can be modulated by changes in the expression of particular genes [50]. It has been reported that changes in miRNA expression occur with human cellular senescence, which is defined as an irreversible decline in cell proliferation [51]. In this context, Noren Hooten et al. [52] analysed the expression of over 800 miRNAs in the blood of younger and older individuals. The authors identified several miRNAs, including miRNA-221 and miRNA-155, that were less expressed in older individuals than in younger individuals. Similarly, Fichtlscherer et al. [53] showed that the age of females was inversely correlated with the circulating miRNA-155 levels in coronary artery disease (CAD) patients and in the healthy population. Furthermore, our results showed that the correlation between miRNA expression and age was lost after RT treatment, which indicated that RT induced an increase in the levels of selected miRNAs, affecting mainly older patients.

We propose that the observed RT-induced modulation of blood miRNA-146a, -155, -221 and -222 might be involved in the progression of CVD and that BC patients usually develop CVD a few years after RT treatment [18,54]. Numerous studies have confirmed that all these miRNAs were overexpressed in atherosclerotic human lesions [32–34]. Thus, it would be expected to find high levels of these miRNAs in the circulation in CVD patients, as they are involved in this pathology. In fact, circulating miRNA-146a was overexpressed in CAD patients [55–57]. However, other studies have reported low levels of miRNA-146a, as well as miRNA-155, -221 and -222, in the circulation of patients with dyslipidaemia, CAD, coronary heart disease (CHD) or atherosclerosis [47,53,56,58,59]. The observed miRNA downregulation in these studies could be explained by the fact that miRNAs can be taken up by atherosclerotic lesions or perhaps a feedback mechanism could control their overactivation. In the current study, to assess the effect of RT on the selected miRNA levels, we compared the expression of each miRNA in the blood between pre-RT control and post-RT samples. We observed significant overexpression of miRNA-146a, -221 and -222 and an increased tendency of miRNA-155 in the blood one month after RT treatment. In previous investigations, miRNA-146a exhibited atheroprotective and anti-inflammatory properties by diminishing lipid uptake and cytokine release by oxidized LDL-activated macrophages [28,35]. Nevertheless, the absence of this miRNA paradoxically suppressed the development of atherosclerosis in mice [32]. On the other hand, it has been reported that miRNA-155 is highly expressed in activated immune cells and regulates immune and inflammatory responses and induces lipid uptake in monocytes and macrophages [29,36]. In a previous investigation carried out by Templin et al. [60], the effect of RT on blood miRNA profiles was analysed. The authors reported 45 differentially expressed miRNAs, including miRNA-221 and -222, that were overexpressed in the blood from patients in complete remission 1 or 2 after 4 hours of total body irradiation at a dose of 1.25 Gy. In contrast, Sochor et al. [61] stated that chemotherapy or RT did not affect the circulating levels of miRNA-155. The discordance between this study and ours could be due to differences between RT treatments, as their patients received two single RT sessions, and post-RT samples were taken at variable time points. Moreover, we observed that miRNA-222 had a higher increase. Although miRNA-221 and -222 contain the same seed [62] and both play a role in endothelial dysfunction and atherogenesis [37,63], miRNA-222 was demonstrated to be more biologically relevant for inflammation-mediated neovessel formation [64]. Therefore, our results, in conjunction with previous data, suggest that selected miRNAs could be overexpressed due to RT in BC patients after one month and might become downregulated many years later when developing CVD because they migrate into atherosclerotic lesions.

One goal of our study was to analyse differences in the expression of selected miRNAs considering CVD-linked clinical parameters, treatment received and BC side. First, we analysed dyslipidaemia and hypothyroidism, as both are well-known risk factors for CVD [65,66]. On the one hand, the results showed that women with BC who were dyslipidaemic had significantly lower expression of selected miRNAs than nondyslipidaemic women both pre-RT control and post-RT. Previous studies have assessed miRNA-146a levels in non-BC patients with dyslipidaemia. For instance, Baldeon et al. [47] found low levels of circulating miRNA-146a in the diabetes population, in which dyslipidaemia is a common feature. In contrast, this miRNA was overexpressed in the circulation in patients with hyperlipidaemia [67]. The disagreements between the results of both studies might be due to differences between the dyslipidaemic profiles of the patients studied. In the same way, our results showed that patients with hypothyroidism exhibited significantly lower miRNA levels than nonhypothyroid patients before and after RT, except for miRNA-221 pre-RT. In contrast, in a recent study enrolling 192 subjects, Quan et al. [68] found that circulating miRNA-146a was higher in patients with CHD and subclinical hypothyroidism than in those with only CHD, suggesting that hypothyroidism was responsible for miRNA-146a overexpression. It should be noted that the results of Quan et al. [68] differed from ours because their study examined not BC but rather CHD patients. Moreover, our results confirm the relationship between selected miRNA downregulation and CVD, as we showed that dyslipidaemic and hypothyroid patients had low levels of selected miRNAs.

On the other hand, we assessed whether treatment could lead to differential miRNA expression under both pre-RT control and post-RT conditions. It has been described that chemotherapy as well as targeted and hormonal therapies are involved in cardiopathologies but exhibit less severity than RT [69–72]. In the present study, some patients received chemotherapy at the pre-RT control time point. Our results established that women with BC who received chemotherapy overexpressed miRNA-146a and -222 pre-RT and miRNA-155 and -221 post-RT compared to those not treated with chemotherapy. Interestingly, other authors have found diminished circulating levels of miRNA-155 and -221 at the end of chemotherapy in BC patients [43,73], whereas Rigaud et al. [74] found increased miRNA-146a levels 3 weeks after the conclusion of chemotherapy in BC patients; the results are in agreement with ours. Furthermore, the differences in the expression of these miRNAs due to chemotherapy, as an established risk of cardiotoxicity [70,72], might support their role in CVD. In addition, our results indicated that blood miRNA-155, -221 and -222 levels were enhanced prior to RT in women who received targeted therapy compared to other women. Therefore, we suppose that this miRNA increase might be related to targeted therapy-associated CVD [69,71]. According to the data obtained in our study, we suggest that hormonal therapy, which is applied at the same time as RT in some women with BC, did not interfere with miRNA expression, because we did not find any differences between the miRNA levels in hormonal therapy-treated and nontreated patients either pre- or post-RT. Furthermore, miRNA-146a was overexpressed in patients with left-sided BC before and after RT and miRNA-221 post-RT when compared to patients with right-sided BC. Thus, a possible link between the observed modulation of miRNA expression and CVD might exist, as RT on left-sided tumours induced greater cardiotoxicity than that on right-sided tumours [10].

In conclusion, our results showed that RT led to the overexpression of miRNA-146a, -155, -221 and -222, which are involved in the progression of CVD, in the blood of women with BC. The expression of each of the miRNAs was positively correlated with that of the others pre-RT control and post-RT and inversely correlated with age pre-RT control. Furthermore, CVD-linked clinical parameters, such as dyslipidaemia and hypothyroidism, as well as treatment with chemotherapy and targeted therapy and BC side, also affected their levels. Thus, the modulation of these miRNAs together with other risk factors might be associated with the development of future cardiovascular pathologies. Variation in the miRNA signature in a diseased peripheral circulatory system opens up a new avenue in the field of biomarker discovery. However, further confirmatory studies are needed to assess their potential in the progression of CVD or as therapeutic targets in the treatment of RT-induced CVD in BC patients.

## Supporting information

S1 Table Correlations between expression of selected miRNAs in blood of BC patients and age at diagnose. (DOCX)

S2 Table Expression of selected miRNAs expression in blood of BC patients at pre-RT control and post-RT depending on RT scheduled and hormonal therapy. BC, breast cancer; RT, radiotherapy; miRNA, microRNA.
Mean ± SEM of the expression (log(2^-DCt^)+3) of selected miRNAs is shown. Statistics: Single factor ANOVA test. p value indicates differences between groups.
BC, breast cancer; RT, radiotherapy; miRNA, microRNA.
miRNA expression was calculated as log(2^-DCt^)+3. Pearson correlation coefficient (r) is shown. **means significant differences at p<0.01 (bilateral).
(DOCX)
