# Supplementary material for: Effect of radiotherapy on the expression of cardiovascular disease-related miRNA-146a, -155, -221 and -222 in blood of women with breast cancer
Source: PLoS One. 2019 May 31;14(5):e0217443. doi: 10.1371/journal.pone.0217443 (PMC6544229; doi:10.1371/journal.pone.0217443)
Supplement: S1 Table — (DOCX) [file pone.0217443.s001.docx]

**Supporting information**

**Table S1. Correlations between expression of selected miRNAs in blood of BC patients and age at diagnose.**

|  | Pre-RT control | | | |
| --- | --- | --- | --- | --- |
|  | miRNA-146a | miRNA-155 | miRNA-221 | miRNA-222 |
| Age at diagnose | -0.130 | -0.103 | -0.141 | -0.148 |
