# Supplementary material for: Effect of radiotherapy on the expression of cardiovascular disease-related miRNA-146a, -155, -221 and -222 in blood of women with breast cancer
Source: PLoS One. 2019 May 31;14(5):e0217443. doi: 10.1371/journal.pone.0217443 (PMC6544229; doi:10.1371/journal.pone.0217443)
Supplement: S2 Table — BC, breast cancer; RT, radiotherapy; miRNA, microRNA. Mean ± SEM of the expression (log(2-DCt)+3) of selected miRNAs is shown. Statistics: Single factor ANOVA test. p value indicates differences between groups. BC, breast cancer; RT, radiotherapy; miRNA, microRNA. miRNA expression was calculated as log(2-DCt)+3. Pearson correlation coefficient (r) is shown. **means significant differences at p<0.01 (bilateral). (DOCX) [file pone.0217443.s002.docx]

**Supporting information**

**Table S2. Expression of selected miRNAs expression in blood of BC patients at pre-RT control and post-RT depending on RT scheduled and hormonal therapy.**

| \|  \| **Pre-RT control** \| \| \| **Post-RT** \| \| \| \| --- \| --- \| --- \| --- \| --- \| --- \| --- \| \| **RT scheduled** \| **Normofractionated**  **(82.35 %)** \| **Hypofractionated**  **(17.65%)** \| **p value** \| **Normofractionated**  **(82.35 %)** \| **Hypofractionated**  **(17.65%)** \| **p value** \| \| **miRNA-146a** \| 2.33±0.039 \| 2.35±0.078 \| 0.785 \| 2.39±0.038 \| 2.46±0.068 \| 0.444 \| \| **miRNA-155** \| 2.04±0.041 \| 2.029±0.082 \| 0.940 \| 2.06±0.040 \| 2.12±0.082 \| 0.514 \| \| **miRNA-221** \| 2.65±0.044 \| 2.68±0.074 \| 0.793 \| 2.74±0.039 \| 2.79±0.085 \| 0.603 \| \| **miRNA-222** \| 3.31±0.045 \| 3.30±0.084 \| 0.939 \| 3.42±0.042 \| 3.45±0.095 \| 0.814 \| \| **Hormonal therapy** \| **No**  **(21.3%)** \| **Yes**  **(78.7%)** \| **p value** \| **No**  **(21.3%)** \| **Yes**  **(78.7%)** \| **p value** \| \| **miRNA-146a** \| 2.25±0.074 \| 2.34±0.041 \| 0.346 \| 2.30±0.090 \| 2.43±0.037 \| 0.151 \| \| **miRNA-155** \| 1.96±0.084 \| 2.05±0.042 \| 0.398 \| 2.07±0.094 \| 2.08±0.041 \| 0.928 \| \| **miRNA-221** \| 2.62±0.090 \| 2.66±0.043 \| 0.716 \| 2.70±0.100 \| 2.76±0.039 \| 0.542 \| \| **miRNA-222** \| 3.26±0.081 \| 3.31±0.045 \| 0.680 \| 3.40±0.119 \| 3.44±0.041 \| 0.698 \| \|  \| \| \| \| \| \| \| |
| --- | --- | --- | --- | --- | --- | --- | --- | --- | --- | --- | --- | --- | --- | --- | --- | --- | --- | --- | --- | --- | --- | --- | --- | --- | --- | --- | --- | --- | --- | --- | --- | --- | --- | --- | --- | --- | --- | --- | --- | --- | --- | --- | --- | --- | --- | --- | --- | --- | --- | --- | --- | --- | --- | --- | --- | --- | --- | --- | --- | --- | --- | --- | --- | --- | --- | --- | --- | --- | --- | --- | --- | --- | --- | --- | --- | --- | --- | --- | --- | --- | --- | --- | --- | --- |

BC, breast cancer; RT, radiotherapy; miRNA, microRNA.

Mean ± SEM of the expression (log(2-DCt)+3) of selected miRNAs is shown. Statistics: Single factor ANOVA test. p value indicates differences between groups.

BC, breast cancer; RT, radiotherapy; miRNA, microRNA.

miRNA expression was calculated as log(2-DCt)+3. Pearson correlation coefficient (r) is shown. **means significant differences at p<0.01 (bilateral).
